# Prediction Model for Lung Cancer in High-Risk Nodules Being Considered for Resection: Development and Validation in a Chinese Population

**DOI:** 10.3389/fonc.2021.700179

**Published:** 2021-09-24

**Authors:** Chunqiu Xia, Minghui Liu, Xin Li, Hongbing Zhang, Xuanguang Li, Di Wu, Dian Ren, Yu Hua, Ming Dong, Hongyu Liu, Jun Chen

**Affiliations:** ^1^ Department of Lung Cancer Surgery, Tianjin Medical University General Hospital, Tianjin, China; ^2^ Tianjin Key Laboratory of Lung Cancer Metastasis and Tumor Microenvironment, Tianjin Lung Cancer Institute, Tianjin Medical University General Hospital, Tianjin, China; ^3^ Department of Thoracic Surgery, First Affiliated Hospital, School of Medicine, Shihezi University, Shihezi, China

**Keywords:** lung cancer, pulmonary nodule, prediction model, clinical decision making, lung surgery

## Abstract

**Background:**

Determining benign and malignant nodules before surgery is very difficult when managing patients with pulmonary nodules, which further makes it difficult to choose an appropriate treatment. This study aimed to develop a lung cancer risk prediction model for predicting the nature of the nodule in patients’ lungs and deciding whether to perform a surgical intervention.

**Methods:**

This retrospective study included patients with pulmonary nodules who underwent lobectomy or sublobectomy at Tianjin Medical University General Hospital between 2017 and 2020. All subjects were further divided into training and validation sets. Multivariable logistic regression models with backward selection based on the Akaike information criterion were used to identify independent predictors and develop prediction models.

**Results:**

To build and validate the model, 503 and 260 malignant and benign nodules were used. Covariates predicting lung cancer in the current model included female sex, age, smoking history, nodule type (pure ground-glass and part-solid), nodule diameter, lobulation, margin (smooth, or spiculated), calcification, intranodular vascularity, pleural indentation, and carcinoembryonic antigen. The final model of this study showed excellent discrimination and calibration with a concordance index (C-index) of 0.914 (0.890–0.939). In an independent sample used for validation, the C-index for the current model was 0.876 (0.825–0.927) compared with 0.644 (0.559–0.728) and 0.681 (0.605–0.757) for the Mayo and Brock models. The decision curve analysis showed that the current model had higher discriminatory power for malignancy than the Mayo and the Brock models.

**Conclusions:**

The current model can be used in estimating the probability of lung cancer in nodules requiring surgical intervention. It may reduce unnecessary procedures for benign nodules and prompt diagnosis and treatment of malignant nodules.

## Introduction

Lung cancer is the most common malignancy in the world and is the highest cause for cancer mortality (1). With a very poor prognosis, the 5-year survival rate for lung cancer is only 19.7% (2, 3) despite recent improvements (4). According to the eighth edition of the TNM staging of lung cancer published by the International Association for the Study of Lung Cancer, 80% of patients with stage IA non–small-cell lung cancer (NSCLC) are alive for ≥5 years after diagnosis. However, this proportion drops to <10% in patients with stage IV disease (5). The poor survival of patients with lung cancer may primarily be due to the fact that the majority of patients are diagnosed at an advanced stage (6).

Based on the findings of the National Lung Screening Trial (7), computed tomography (CT) or low-dose CT (LDCT) has been recommended as an effective tool for lung cancer screening in many countries or regions (8–11). Although CT or LDCT helps detect lung cancer at an early stage, the majority of pulmonary nodules (PNs) detected by CT are benign (7). Identifying malignant PNs from benign ones has become a challenge for clinicians, and follow-up examinations (e.g., follow-up scans and invasive biopsies) may lead to additional costs or harm the patient (12). In recent decades, several lung cancer risk prediction models based on radiological characteristics and clinical information have been developed to assist clinicians in managing patients with pulmonary nodules (13–18). These models have demonstrated a high value in discriminating independent cohorts. Moreover, some of them were recommended by guidelines for the classification of high- and low-risk pulmonary nodules (11).

However, most of these models were built on initial CT plain or LDCT scans and were used at the baseline. However, the diagnostic performance of models may be inaccurate within dissimilar populations. Clinicians rarely recommend performing an invasive procedure in patients with PNs after their initial scan. Consequently, a period of observation for PNs often exists before they make a decision. Having a tool accurate enough to assist clinicians in judging would be clinically useful to help avoid overdiagnosis and facilitate early diagnosis before deciding on an invasive procedure. Different from the previously reported models, the subjects of this study were those with PNs that were highly suspected by clinicians to be lung cancer (all of these patients underwent surgery).

## Patients and Methods

### Study Population

The training database included a retrospective sample of patients with at least one pulmonary nodule diameter ranging from 5 to 30 mm on CT lung window with a definitive histopathologic diagnosis by surgery at Tianjin Medical University General Hospital between 2017 and 2019. Individuals with atelectasis, obstructive pneumonia, or pleural effusion on CT; ongoing antitumor therapy; preoperative non-surgical histopathologic diagnosis; history of lung cancer diagnosis; history of pulmonary surgery; pulmonary metastatic disease; and age < 18 years were excluded. Patient and clinicopathologic characteristics were collected through chart review and electronic medical records. A malignant or benign diagnosis was established by pathologic tissue examination *via* complete nodule resection or the lobe it resides (including lobectomy and sublobectomy). The validation dataset included individuals with the same criteria diagnosed between 2019 and 2020 and was collected independently of the training cohort. There were 785 patients who met the inclusion criteria. Twenty-two patients were excluded because of the lack of CT data. Eventually, 763 patients were enrolled in this study. The model training set included the contrast-enhanced preoperative CT images of the patients.

Conventional radiologic staging before surgery generally includes contrast-enhanced CT of the chest and abdomen, emission computed tomography of bone, and magnetic resonance imaging of brain. Clinical data collection, shown in **Tables 1**–**3** according to lung cancer status, included clinical characteristics, radiographic PN characteristics, and serum tumor markers [carcinoembryonic antigen (CEA), cytokeratin fraction 21-1 (CYFRA 21-1), squamous cell carcinoma antigen (SCC), and neuron-specific enolase (NSE)]. Clinical characteristics included sex, age at diagnosis, smoking history, cancer history other than lung cancer, and family history of lung cancer. Two experienced thoracic radiologists identified and characterized PNs according to lobar location, size (long-axis diameter), presence (e.g., spiculation, calcification, and lobulation), and type (ground-glass, part-solid, or solid nodules). Nodules would be characterized as multiple if more than one similar nodule exist and are considered to be the same disease. Lymphadenopathy was defined as lymph nodes in pulmonary or mediastinum of >10 mm in short-axis diameter.

**Table 1:** Demographic characteristics according to lung cancer status in the study and validation datasets.

Variables	Study group (N = 563)			Validation group (N = 200)		
	Benign (N = 193)	Malignant (N= 370)	p value	Benign (N = 67)	Malignant (N = 133)	p value
**Age at diagnosis (years), mean ± SD**	57.6 ± 10.5	63.0 ± 8.6	<0.001	57.9 ± 11.2	61.5 ± 8.8	0.012
**Gender, n (%)**			0.075			0.073
**Male**	102 (52.8)	165 (44.6)		38 (56.7)	57 (42.9)	
**Female**	91 (47.2)	205 (55.4)		29 (43.3)	76 (57.1)	
**Family history of lung cancer, n (%)**	9 (0.05)	30 (8.1)	0.162	0 (0.0)	2 (1.5)	0.552
**History of cancer other than lung, n (%)**	7 (3.6)	25 (6.8)	0.178	2 (3.0)	2 (1.5)	0.603
**Active or former smoker, n (%)**	72 (37.3)	141 (38.1)	0.927	28 (47.8)	47 (35.3)	0.439
**Emphysema or COPD, n (%)**	36 (18.7)	76 (20.5)	0.657	19 (28.4)	24 (18.0)	0.103

### Statistical Analysis

Descriptive statistics were used to describe the characteristics of the patient cohorts. Continuous data were expressed as means ± standard deviation or median with interquartile ranges and were compared between groups using the Student’s *t*-test or the Mann–Whitney *U* test, as appropriate. Categorical data were given as counts and percentages and were analyzed using Pearson *χ* ^2^ tests. Binomial logistic regression models were used, and the Akaike information criterion values were applied to determine which combinations of model predictors best explain the data. Model performance was assessed using estimates of discrimination (ability to classify benign and malignant PNs) and calibration (how well probabilities predicted by the model agree with actual observed risk). The Harrell C-index measures discrimination and is corrected using 1,000 bootstrap resamples (19). Calibration was assessed by plotting the subtraction of actual (Kaplan–Meier method) and predicted survival probabilities of malignancy (20, 21). The area under the receiver operating characteristic curve (AUC) values and decision curve analysis (DCA) (22) were used to assess the diagnostic performance of all models. All analyses were two-tailed at a significance level of *p* < 0.05. All statistics were performed with R version 4.0.3 (The R Foundation for Statistical Computing) and SPSS version 23 for Windows.

## Results

### Histopathological Results of Nodules

Of the patients, 563 were identified for model building. Moreover, 370 (65.7%) of the patients had malignant PNs. Among the malignant PNs (503 individuals) in both study and validation groups, 424 (84.3%), 45 (8.9%), 13 (2.6%), 7 (1.4%), 6 (1.2%), 5 (1.0%), and 3 (0.6%) were adenocarcinomas, squamous cell carcinomas, cancer *in situ*, large cell carcinomas, carcinoid tumors, small cell carcinomas, and other malignant histologies, respectively. According to the eighth edition of the TNM staging system, 13 of NSCLCs were Tis; 307 of NSCLCs were T1N0M0, 100 of them were T1a(mi), 14 of them were T1a, 156 of them were T1b, and 37 of them were T1c; 175 of NSCLC were T2aN0-1M0 (with invasion visceral pleural, or involvement of main bronchus without carina); and 27 of them were N1 stage. All five small cell lung cancers were limited stage. Of the benign PNs, 110 (42.3%), 46 (17.7%), 45 (17.3%), 14 (5.4%), 13 (5.0%), 11 (4.2%), and 21 (8.1%) were granulomas (including inflammatory pseudotumor, tuberculosis, pulmonary mycosis, and melioidosis), pneumonia or organizing pneumonia, hamartomas, sclerosing pneumocytoma, lymph nodes, atypical adenomatous hyperplasia, and other benign histologies, respectively.

### Clinical and Nodule Characteristic

The patients in the malignant group were older (57.6 ± 10.5 vs. 63.0 ± 8.6, *p* < 0.001), and malignant nodules were more frequent in females than males (55.4% vs. 44.6%; *p* = 0.075). Of the patients, 213 (37.8%) and 36 (5.7%) were current or former smokers and had a history of extrathoracic cancer, respectively. Moreover, 112 (19.9%) patients had a history of chronic obstructive pulmonary disease (COPD) or radiographic evidence of emphysema. The clinical characteristics of patients are shown in **Table 1**.

The majority of PNs were solid (347, 61.6%). The frequency of malignancy was significantly higher in subsolid nodules (part-solid nodules and ground-glass nodules) than in the solid nodules (90.5% vs. 87.4% vs. 51.3%, respectively; *p* < 0.001). The median nodule diameter was 14.0 mm (interquartile range, 10.0–20.0 mm) and 17.0 mm (interquartile range, 12.0–22.0 mm) for benign and malignant (*p* = 0.001), respectively. Malignant nodules were more likely located in the upper lobe than the other lobes (62.2% vs. 37.8%, *p* = 0.001). Nodules with lymphadenopathy, lobulation, spiculation, vacuole sign or air bronchogram, pleural indentation, and internal vascularity have a higher proportion of malignancy. The CT characteristics of nodules are described in **Table 2**.

**Table 2:** CT characteristics of the nodules according to lung cancer status in the study and validation datasets.

Variables	Study group (N = 563)			Validation group (N = 200)		
	Benign (N = 193)	Malignant (N = 370)	p value	Benign (N= 67)	Malignant (N= 133)	p value
**Nodule location, n (%)**						
**Left lobe**	78 (40.4)	143 (38.6)	0.716	25 (37.3)	50 (37.6)	>0.9
**Right lobe**	115 (59.6)	227 (61.4)		42 (62.7)	83 (62.4)	
**Upper lobe**	91 (47.2)	230 (62.2)	0.001	27 (40.3)	81 (60.9)	0.007
**Middle or lower lobe**	102 (52.8)	140 (37.8)		40 (59.7)	52 (39.1)	
**Lymphadenopathy, n (%)**	26 (13.5)	73 (19.7)	0.08	6 (9.0)	25 (18.8)	0.097
**Solitary nodule, n (%)**	156 (80.8)	288 (77.8)	0.447	52 (77.6)	101 (75.9)	0.861
**Multiple nodules, n (%)**	37 (19.2)	82 (22.2)		15 (22.4)	32 (24.1)	
**Nodule type, n (%)**			<0.001			<0.001
**Solid**	169 (87.6)	178 (48.1)		45 (67.2)	50 (37.6)	
**Part-solid**	10 (5.2)	95 (25.7)		10 (14.9)	42 (31.6)	
**Nonsolid**	14 (7.3)	97 (26.2)		12 (17.9)	41 (30.8)	
**Margin, n (%)**						
**Smooth**	65 (33.7)	40 (10.8)	<0.001	19 (28.4)	4 (3.0)	<0.001
**Spiculated**	38 (19.7)	149 (40.3)	<0.001	15 (22.4)	54 (40.6)	0.012
**Lobulation, n (%)**	27 (14.0)	89 (24.1)	0.006	10 (14.9)	23 (17.3)	0.84
**Calcification, n (%)**	22 (11.4)	3 (0.8)	<0.001	5 (7.5)	1 (0.8)	0.017
**Pleural indentation, n (%)**	24 (12.4)	136 (36.8)	<0.001	6 (9.0)	57 (42.9)	<0.001
**Nodule diameter (mm), median (IQR)**	14.0 (10.0–20.0)	17.0 (12.0–22.0)	0.001	13.0 (9.0–20.0)	16.0 (11.5–20.0)	0.028
**Vacuole or air Bronchogram, n (%)**	47 (24.4)	152 (41.1)	<0.001	13 (19.4)	51 (38.3)	0.007
**Intranodular vascularity, n (%)**	80 (41.5)	319 (86.2)	<0.001	13 (19.4)	95 (71.4)	<0.001

Of the patients, 197 (35.0%) had at least one tumor marker elevated at diagnosis, and 138 of them were malignant. Median CEA and CYFRA 21-1 in malignant nodules were significantly (*p* < 0.05) higher than those in benign nodules. The serum tumor markers of patients are summarized in **Table 3**.

**Table 3:** Serum tumor markers according to lung cancer status in the study and validation datasets.

Variables	Study group (N = 563)			Validation group (N = 200)		
	Benign (N = 193)	Malignant (N = 370)	p value	Benign (N = 67)	Malignant (N= 133)	p value
**CEA (ng/ml)**						
**Median (IQR)**	1.96 (1.40–3.07)	2.44 (1.55–3.65)	0.002	2.04 (1.33–2.07)	2.26 (1.57–3.39)	0.253
**Elevated, n (%)**	12 (6.2)	44 (11.9)	0.037	9 (13.4)	14 (10.5)	0.639
**CYFRA 21-1 (ng/ml)**						
**Median (IQR)**	1.74 (1.31–2.36)	2.04 (1.48–2.78)	<0.001	1.65 (1.14–2.32)	1.88 (1.46–2.42)	0.039
**Elevated, n (%)**	14 (7.3)	44 (11.9)	0.108	7 (10.4)	15 (11.3)	>0.9
**SCC (μg/L)**						
**Median (IQR)**	0.7 (0.5–1.0)	0.7 (0.5–1.0)	0.313	0.8 (0.5–1.0)	0.7 (0.6–1.0)	0.624
**Elevated, n (%)**	15 (7.8)	29 (7.8)	>0.9	6 (9.0)	8 (6.0)	0.558
**NSE (ng/ml)**						
**Median (IQR)**	12.71 (10.54–15.41)	12.1 (10.47–14.47)	0.415	13.89 (11.51–15.60)	14.19 (11.90–17.20)	0.195
**Elevated, n (%)**	35 (18.1)	49 (13.2)	0.135	14 (20.9)	36 (27.1)	0.39
**Elevated of tumor markers, n (%)**			0.115			0.286
**None**	134 (69.4)	232 (62.7)		44 (65.7)	76 (57.1)	
**One or more**	59 (30.6)	138 (37.3)		23 (34.3)	57 (42.9)	

CEA > 5.0 ng/ml, CYFRA 21–1 >3.3 ng/ml, SCCA >1.5 μg/l, and NSE > 16 ng/ml were set as elevated tumor markers.

### Predictive Model

**Figure 1:**
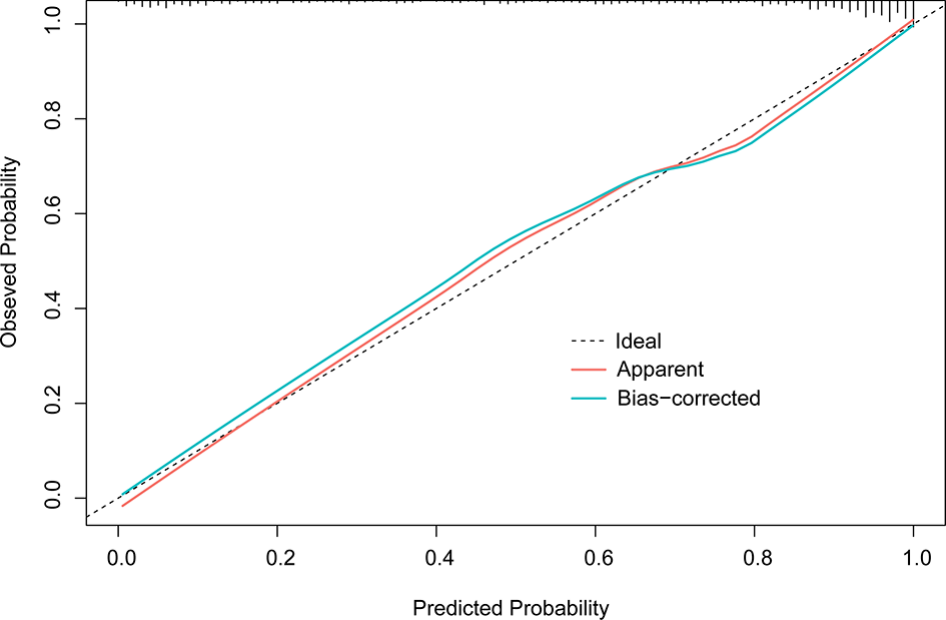
Calibration plot of M1 (1,000 times with bootstrap validation). The ideal line, a 45° *straight dotted line*, illustrates a perfect fit. The apparent and bias-corrected lines are based on the M1 predicted probability and predicted probabilities of bootstrapped samples, respectively.

In the final multivariate logistic regression model (M1), the diagnosis of cancer in a nodule was associated with sex, age at diagnosis, smoking history, lymphadenopathy, vacuole or air bronchogram, nodule type (pure ground-glass and part-solid), nodule diameter, lobulation, margin (smooth, spiculated, or none of these), calcification, intranodular vascularity, pleural indentation, and CEA **Table 4**. M1 showed a highly discriminant ability with a C-index of 0.914 (0.890–0.939) and 0.906 (0.885–0.927) by internal validation with 1,000 times bootstrap resampling and adjustment for optimism. Moreover, the calibration curve for the model is plotted in **Figure 1**.

**Table 4:** Characteristics for covariates in the final model (M1) for the probability of lung cancer in pulmonary nodules.

Covariates	β-coefficient	SE	OR (95% CI)	**p value**
**Intercept**	-6.65670			
**Age (years)**	0.05145	0.01475	1.05 (1.02–1.08)	<0.001
**Gender (male)**	-1.07702	0.34813	0.34 (0.17–0.67)	0.002
**Family history of lung cancer**	0.91006	0.56018	2.48 (0.86–7.87)	0.104
**History of cancer other than lung**	0.99997	0.66999	2.72 (0.80–11.33)	0.136
**Active or former smoker**	0.78488	0.35239	2.19 (1.11–4.43)	0.026
**Nodule type (solid as reference)**				
**Part-solid**	3.11616	0.46168	22.56 (9.58–59.11)	<0.001
**Nonsolid**	3.27864	0.42573	26.54 (11.91–63.57)	<0.001
**Margin**				
**Smooth**	-0.74754	0.37480	0.47 (0.23–0.99)	0.046
**Spiculated**	0.80414	0.32533	2.23 (1.19–4.26)	0.013
**Lobulation**	1.53012	0.36872	4.62 (2.29–9.77)	<0.001
**Calcification**	-2.51902	0.72417	0.08 (0.02–0.29)	<0.001
**Pleural indentation**	1.18775	0.34884	3.28 (1.68–6.63)	<0.001
**Nodule diameter (mm)**	0.04386	0.02143	1.04 (1.00–1.09)	0.041
**Intranodular vascularity**	1.58163	0.28340	4.86 (2.81–8.55)	<0.001
**CEA (ng/ml)**	0.14964	0.06719	1.16 (1.03–1.33)	0.026
**CYFRA 21-1 (ng/ml)**	0.20759	0.13706	1.23 (0.94–1.62)	0.130

A model containing only solid nodules in the training cohort (M2) was subsequently built because of the similar distributions of the benign and malignant PNs. This model reached a C-index of 0.918 (0.890–0.946) and 0.906 (0.874–0.938) after bootstrap validation. Following external validation by solid nodules in the validation set, M1 and M2 produced a C-index of 0.904 (0.847–0.960) and 0.896 (0.836–0.955), respectively. However, these differences were not statistically significant.

### Model Comparison in the Validation Cohort

**Figure 2:**
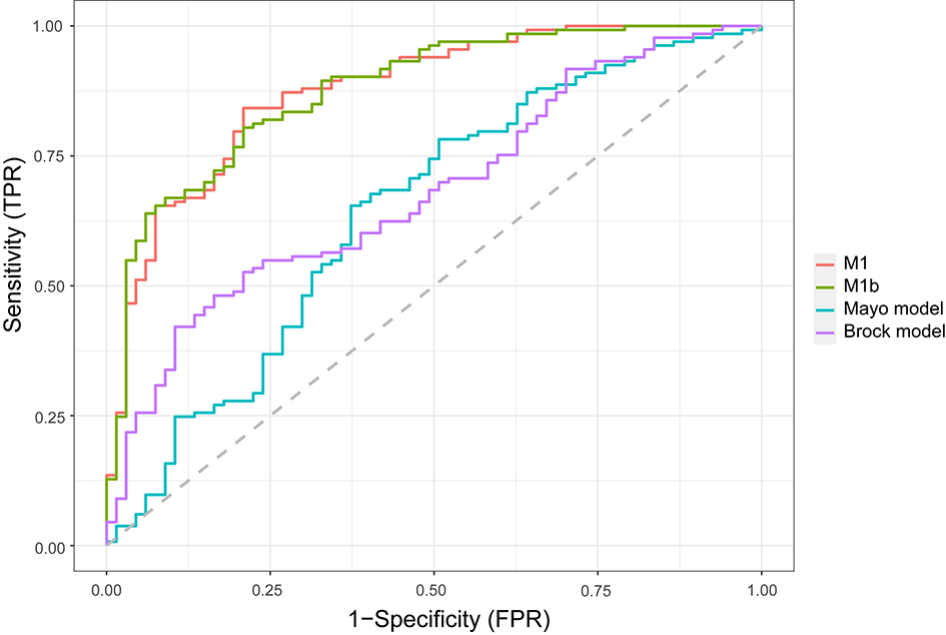
Comparison of lung cancer prediction models in the validation cohort. Model discrimination is measured by area under the ROC curve. *TPR*, true-positive rate; *FPR*, false-positive rate.

In the external validation cohort (**Figure 2**), the diagnostic performance between M1, M1b (M1 without serum tumor markers), Mayo model, and Brock model was compared using AUC, (95% CI). For M1, M1b, Mayo model, and Brock model, the AUC was 0.876 (0.825–0.927), 0.877 (0.827–0.927), 0.644 (0.559–0.728), and 0.681 (0.605–0.757), respectively. The discrimination performance of the current model was significantly better than that of the Mayo (*p* < 0.01) or Brock (*p* < 0.01) models. Notably, the multivariate logistic regression analyses showed that CEA was the independent predictor of malignant nodules, but M1 was not superior to M1b in external validation.

**Figure 3:**
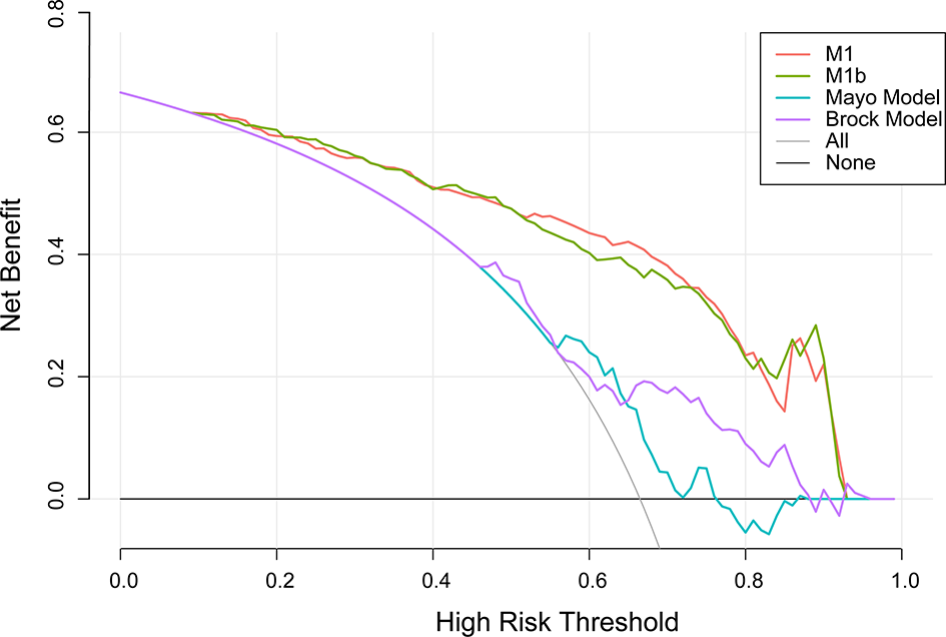
Decision curve analysis for lung cancer prediction models in the validation cohort. *Thick gray oblique line* the strategy of treating all patients; *thick black horizontal line* the strategy of treating no patients. The line with the highest net benefit at a specific threshold probability will lead to the best clinical outcome.

A decision curve (22) was plotted to compare the benefit of these three models, and these results were put in a clinical context (**Figure 3**). The net benefit of M1 was better than either the Mayo or Brock models for all threshold probabilities of >10% in clinical settings. Thus, patients whose cancer risk was approximately one in 10 or higher and who receive surgery would benefit from the current model.

**Figure 4:**
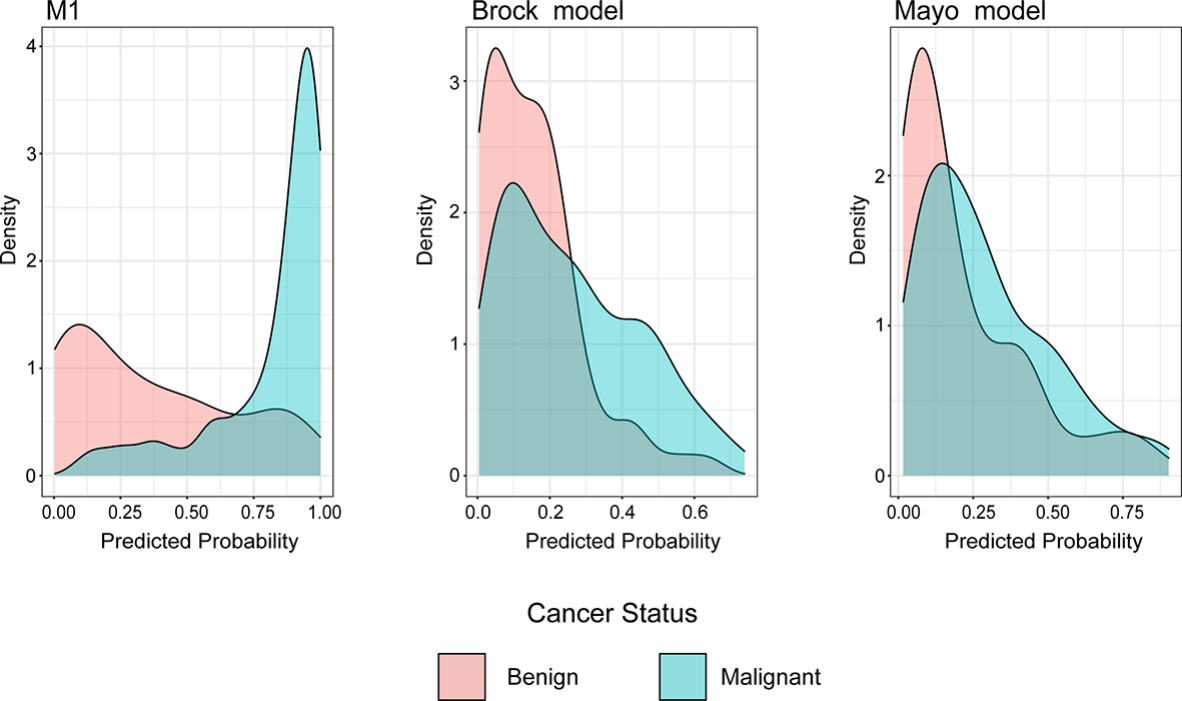
Distributions of predicted lung cancer probability across models for patients with malignant and benign nodules in the validation cohort.

The density distribution of the predicted probability score on the validation cohort of three models is shown in **Figure 4**. The M1 score was >75% for 79% of individuals with malignant PNs, whereas subjects with benign PNs tend to be distributed. In contrast, the Mayo or Brock models have insignificant concentration trends.

## Discussion

Early detection and accurate diagnosis are effective ways to lower lung cancer mortality. Given the occult onset, CT screening may be currently the preferred test for early diagnosis and management of clinically significant lung nodules. However, the optimal target PNs and the timing of biopsy remain uncertain (23). The American College of Chest Physicians (CHEST) guidelines for lung cancer screening (version 2021) summarized the results of 17 clinical trials and revealed that 22.0% of surgeries were performed for benign diseases (ranged from 8% to 39%) (24). How to reduce benign resection without delaying the diagnosis of lung cancer has become a research hotspot. This evidence-based, retrospective project established a malignancy risk prediction model to reassess the PNs that clinicians considered need to be biopsied. This study reviewed data from 763 subjects diagnosed with lung nodules that were clinically considered to be highly malignant who underwent surgical resection in between 2017 and 2020. Except for a few confirmed benign diseases, most nodules were considered to be malignant preoperatively. Despite the received observation and intervention recommended in the guidelines (11, 25, 26) before surgery, nearly one in three nodules remained benign. The current initial M1, built with all predictors, showed excellent predictive accuracy (with an AUC of 0.876 in an external validation cohort) and calibration (**Figure 1**). M2 was built because of the difference in the distributions of benign and malignant lesions in three nodule densities. However, M2 did not perform better in classifying solid nodules in the validation cohort (AUC, 0.904 vs. 0.896) than M1. Serum tumor markers did not prove to be a strong predictor as anticipated in the multivariate analyses. Thus, the M1b model was built to exclude tumor markers. In the validation data, which tend to be lower tumor markers levels even when malignant, M1 did not perform better than M1b. Even if CEA levels show differences between benign and malignant nodules, the effectiveness of tumor markers in the classification of PNs needs further verification.

Smoking is a risk factor for lung cancer (13, 14, 27). The smoking rate of malignant cohort in this study was 37% which was much lower than that in other studies, especially screen-based studies (15, 28, 29). Moreover, smoking history was an independent predictor for lung cancer in the current final multivariable model although no difference was demonstrated in the groups. The smoking prevalence in the current study may be lower greatly because of the varying smoking habits in the male and female populations (30). Females had a lower smoking prevalence than males in this study (10.2% vs. 68.2%, p < 0.001). Moreover, females were significantly associated with malignant PNs, which agrees with previous studies (15, 16, 31). Emphysema or COPD had been noticed to increase the risk of lung cancer (32), but it was not observed in this study. An intranodular vascularity was found to strongly correlate with lung cancer risk, which is consistent with the theory of tumor angiogenesis (33). Malignancy proportion was more frequent in subsolid nodules than in solid nodules because most subsolid nodules resected in this study were monitored until change in follow-up CT features. However, this process may exclude some benign lesions. Changes in CT image of subsolid PNs suggest malignancy (34, 35). Although the largest in diameter did not mean the highest probability of malignancy (15), similar to previous studies (13, 14, 18), malignancies were more often found in bigger nodules in our study (17 mm vs. 14 mm, p = 0.001). Other risk factors for earlier lung cancer differential diagnosis (e.g., nodules with spiculation, lobulation, calcification, or pleural indentation) were also significantly associated with lung cancer in this study (36–38).

Unlike previous models (13–18), the current model was determined following the preoperative contrast-enhanced CT scan and serum tumor markers. In the external validation set, the AUC for the current models was 0.876 compared with 0.644 and 0.683 for the Mayo and Brock models (**Figure 2**). These models were also compared using the decision curve (**Figure 3**), which showed that the current model had higher discriminatory power for malignancy than the Mayo or the Brock model. The density distribution of the predicted probability score of these models on the validation set was plotted to figure out whether these differences would be helpful in the clinical management of patients with PNs with a risk that is high enough to have an invasive procedure (**Figure 4**). The current model classified 79% and 2% of malignant nodules at a probability threshold of ≥0.75 and ≤0.25, respectively. In comparison, the Mayo and Brock models have skewed score distributions for all PNs. Although the current model gave values for discrimination that outperforms the Mayo or the Brock model, they cannot be directly compared because accuracy can considerably vary within populations (39). The malignancy proportion of the Mayo (23.2%) and Brock (5.5%) models is much lower than that of the patients whose PNs were suspected to be malignant after observation recommended by guidelines. The models derived from the populations with a low prevalence of malignancy may underestimate the risk when used in the high-prevalence populations. Therefore, we suggest that medical centers could develop models according to their local populations to help with the clinical management of PNs, instead of directly applying some screening models. The current model is more suitable for reassessment for patients who were admitted for planned surgery or biopsy. The proportion of malignant and benign nodules in the density distribution of the predicted probability of the current model may be helpful in clinical decision-making given the pros and cons of observation, biopsy, or surgery (**Figure 4**).

This study has several limitations. First, the history of previous imaging follow-up of the patient cohort was incomplete as ours was a tertiary referral center. Therefore, this study was unable to evaluate the effect of temporal nodule evolution. Moreover, there was a lack of uniform criteria for suspicion of malignancy, and they were determined based on the subjective judgment of thoracic surgeons. Furthermore, the time point to split the data into study and validation cohorts was used to limit the effect of overfitting. The current model may not perform as well in other study populations. Second, this study failed to build a model exclusively for subsolid nodules. The proportion of benign lesions was only 1 in 10 for subsolid nodules in this study and was too low to perform a multivariate logistic regression. The most likely explanation is that the subsolid nodules included in this study were all observed until they change in follow-up CT features. The changes were suspected to demonstrate usefulness in discriminating benign from malignant nodules. Unfortunately, however, we failed to sum up the period. Lastly, this study was not able to examine nodule classification models that incorporated other factors associated with lung cancer risk [i.e., positron emission tomography-CT (40) and nodule volume (16, 41)] due to the lack of such data.

## Conclusions

This study developed and externally validated a risk model for estimating the probability of lung cancer in PNs that were recommended to have invasive interventions. The model could be considered before more invasive treatments to justify the necessity. Established by using readily available clinical information, this model provides valuable data for clinicians in decision-making. However, the application of the current model in identifying nodules in other populations, such as a screening population, needs further study.

## Data Availability Statement

The raw data supporting the conclusions of this article will be made available by the authors, without undue reservation.

## Ethics Statement

The authors are accountable for all aspects of the work in ensuring that questions related to the accuracy or integrity of any part of the work are appropriately investigated and resolved. The study was conducted in accordance with the Declaration of Helsinki (as revised in 2013). The study was approved by the institutional ethics committee board of Tianjin Medical University General Hospital (No. IRB2019-KY-153), and individual consent for this retrospective analysis was waived.

## Author Contributions

Study design: CX, ML, and JC. Data acquisition: XiL, DW, YH, and DR. Quality control of data: CX, JC, XiL, MD, and HZ. Data analysis and interpretation: CX, JC, ML, HL, and XuL. Statistical analysis: CX, ML, JC, HZ. Manuscript preparation: CX, JC, ML, XiL, and HL. Manuscript editing and reviewing: all authors. All authors contributed to the article and approved the submitted version.

## Funding

This study was supported by grants from the National Natural Science Foundation of China (82072595, 81773207, and 61973232), Natural Science Foundation of Tianjin (18PTZWHZ00240, 19YFZCSY00040, and 19JCYBJC27000), Shihezi University Oasis Scholars Research Startup Project (LX202002) and Special Support Program for the High Tech Leader and Team of Tianjin (TJTZJH-GCCCXCYTD-2-6), Tianjin Municipal Education Commission Natural Science Foundation (2019KJ202, 2020KJ151), and Tianjin Medical University General Hospital Incubation Fund (ZYYFY2017034). The funding sources had no role in the study design, data collection, and analysis; the decision to publish; or the preparation of the manuscript.

## Conflict of Interest

The authors declare that the research was conducted in the absence of any commercial or financial relationships that could be construed as a potential conflict of interest.

## Publisher’s Note

All claims expressed in this article are solely those of the authors and do not necessarily represent those of their affiliated organizations, or those of the publisher, the editors and the reviewers. Any product that may be evaluated in this article, or claim that may be made by its manufacturer, is not guaranteed or endorsed by the publisher.
